# Bifidobacteria shape host neural circuits during postnatal development by promoting synapse formation and microglial function

**DOI:** 10.1038/s41598-020-64173-3

**Published:** 2020-05-08

**Authors:** Berkley Luck, Melinda A. Engevik, Bhanu Priya Ganesh, Elizabeth P. Lackey, Tao Lin, Miriam Balderas, Angela Major, Jessica Runge, Ruth Ann Luna, Roy V. Sillitoe, James Versalovic

**Affiliations:** 1 Department of Pathology, Texas Children’s Hospital, Houston, Texas United States of America; 2 Department of Pathology & Immunology, Baylor College of Medicine, Houston, Texas United States of America; 3 Department of Neurology, University of Texas Health Science Center, Houston, Texas United States of America; 4 Department of Neuroscience, Baylor College of Medicine, Houston, Texas United States of America; 5 Program in Developmental Biology, Baylor College of Medicine, Houston, Texas United States of America; 6 Texas Children’s Microbiome Center, Texas Children’s Hospital, Houston, Texas United States of America; 7 Integrative Molecular and Biomedical Sciences (IMBS), Baylor College of Medicine, Houston, Texas United States of America

**Keywords:** Microbiota, Neurology

## Abstract

We hypothesized that early-life gut microbiota support the functional organization of neural circuitry in the brain via regulation of synaptic gene expression and modulation of microglial functionality. Germ-free mice were colonized as neonates with either a simplified human infant microbiota consortium consisting of four *Bifidobacterium* species, or with a complex, conventional murine microbiota. We examined the cerebellum, cortex, and hippocampus of both groups of colonized mice in addition to germ-free control mice. At postnatal day 4 (P4), conventionalized mice and *Bifidobacterium*-colonized mice exhibited decreased expression of synapse-promoting genes and increased markers indicative of reactive microglia in the cerebellum, cortex and hippocampus relative to germ-free mice. By P20, both conventional and *Bifidobacterium*-treated mice exhibited normal synaptic density and neuronal activity as measured by density of VGLUT2^+^ puncta and Purkinje cell firing rate respectively, in contrast to the increased synaptic density and decreased firing rate observed in germ-free mice. The conclusions from this study further reveal how bifidobacteria participate in establishing functional neural circuits. Collectively, these data indicate that neonatal microbial colonization of the gut elicits concomitant effects on the host CNS, which promote the homeostatic developmental balance of neural connections during the postnatal time period.

## Introduction

Recent studies have established that members of the intestinal microbiota communicate with the host central nervous system (CNS) and can alter CNS gene expression, neurotransmitter function, and behavior in adult rodents^1,2^. However, we do not yet fully understand the mechanisms by which gut microbes communicate with the brain. This is due in part to the fact that the majority of current work has utilized only complex microbial communities. The presence of complex microbiota obscures which species may be key in driving CNS alterations and makes it difficult to decipher which pathways are selectively activated. As a result, it is difficult to understand the changes in the molecular pathways/cues mediating the observed gut to brain effects. Another caveat of existing work is the use of adult animals, which inherently no longer possess a microbiome resembling that of a postnatal animal (standardly defined as the 6 weeks following birth in humans, and the 21 days following birth in mice). In addition, the matured brain of adult animal models does not accurately replicate the plastic and rapidly changing brain of a postnatal animal. Using adult animals to model the microbiota-gut-brain axis therefore limits the ability to evaluate the impact of the early-life microbiome on CNS development. More precise models need to be developed due to these shortcomings.

Colonization of the intestinal microbiota coincides with the organization of fundamental neural circuitry in the brain during the postnatal period^3,4^. Therefore, the interactions between microbes and host during a limited window of this postnatal timeframe is likely crucial for proper neuronal development. Furthermore, since the infant brain is highly susceptible to external influences during the process of neural circuit wiring and refinement^5^, microbe-driven CNS alterations are predicted to have significant and distinct consequences during early postnatal development. The genus *Bifidobacterium* (phylum Actinobacteria) dominates the human infant intestinal microbiome (~80%)^6–13^. This dominance in early life stands in contrast to the limited bifidobacterial populations present in the adult human gut microbiota (~2-14%)^14–19^. Additionally, the species of bifidobacteria in the gut change with age: *B. longum*, *B. breve*, and *B. bifidum* are frequently present in greater abundances in infants, while *B. catenulatum* and *B. adolescentis* are more prevalent in adults^15,20,21^. Thus, when modeling the microbiome-gut-brain interactions in neonates, the introduction of an adult gut microbiota to germ-free mice may not appropriately recapitulate effects of a bifidobacteria-dominated neonatal microbiome on neurodevelopment^22,23^. We have previously demonstrated that adult mice with a complete native gut microbiota, or “conventionalized” mice, harbor only 12.6 ± 1.8 % *Bifidobacterium*^24^. As a result, we propose that this microbial community is not well suited to reflect the *Bifidobacterium*-dominated microbiome that occurs during human postnatal development.

Studies in adult mouse models have shown that “infant-type” *Bifidobacterium* species exert neuromodulatory effects on the host including altered neuronal firing properties, expression of neurotransmitters and neurotransmitter receptors, and neuronal gene expression profiles, making these species ideal for examination of microbiota-gut-brain communication early in life^25–31^. To address the impact of early-life microbes on CNS development, we colonized neonatal germ-free mice with a consortium of human-derived *Bifidobacterium* strains known to be predominant in the intestines of healthy human infants, but relatively diminished in adults^7,11,15^. We hypothesized that colonization with these specific early-life microbes would have a profound effect on neurodevelopment. These “infant-type” strains were *B. longum* subsp. *infantis, B. breve*, *B. bifidum*, and *B. dentium*. To the best of our knowledge, no previous studies have examined postnatal *Bifidobacterium*-specific effects on neural development.

This study leveraged the cerebellum of postnatally colonized (gnotobiotic) mice as a novel model to examine neural circuit modifications in response to microbial colonization. The cerebellum is a useful brain region to study microbiota-mediated alterations to synaptic functionality and circuit development for several reasons. First, cerebellar development extends well after birth in mice^32^, overlapping in time with the rapid onset of gut microbial colonization. In mice, postnatal day 4 (P4) represents an early stage of postnatal neuronal migration and circuit formation, P10 corresponds to the mid-stage of synaptic reorganization and pruning, and P20 represents the final period of granule cell migration in the cerebellum. Thus, this 20-day postnatal period is attractive for tracking how circuit development and neuronal function may be altered by the postnatal microbiota. In addition, previous studies in adult mice suggest that gene expression and neuronal activity in the cerebellum are modulated by the intestinal microbiota^1,32,33^. Thus, assessing alterations in gene expression may provide further insight on how the microbiota can modulate synapse density, neuronal morphology, and/or connectivity. Finally, the well-described circuit architecture and *in vivo* neuronal firing properties make the cerebellum ideal for testing the functional impact of gut microbiota on neural development. In addition to the cerebellum, we also included the cortex and hippocampus in our studies to achieve a more global view of the impact of postnatal microbial colonization on neurodevelopment.

In the studies described, we examined bacterial-driven alterations in synaptic gene expression and microglial function during postnatal neurodevelopment. We utilized three groups: (1) germ-free mice treated with sterile medium (germ-free; GF), (2) germ-free mice colonized by a defined *Bifidobacterium* consortium (*B. longum* subsp. *infantis, B. breve*, *B. bifidum*, and *B. dentium;* BIF) or (3) germ-free mice colonized by a complex fecal microbial community from specific-pathogen free mice (CONV). All groups were treated/colonized from birth and separately maintained in sterile isolators for the duration of the experiment. The defined microbial consortium of “infant-type” *Bifidobacterium* was designed to model the healthy, human infant microbiota during the postnatal period of human development and we have previously shown that these species are sufficient to prevent the development of abnormal behaviors observed in germ-free adults^24^. Compared to the GF mice, those colonized with both a conventional murine microbiota (CONV mice) and *bifidobacteria* (BIF mice) exhibited diminished expression of synapse-promoting genes, suggesting that these specific genes are overexpressed when microbial signaling is absent. The GF mice also displayed stunted microglial reactivity at P4 relative to BIF and CONV mice. The observed synaptic deficits (both morphological and functional) in GF mice were not observed in BIF or CONV groups, suggesting that neonatal microbial colonization prevents or mitigates these effects. Based on these data, we propose a model by which postnatal microbial colonization promotes network refinement and functional organization of neural circuitry by down-regulating early expression of synapse-promoting genes and promoting the phagocytic activity of microglia. A deeper understanding of host-microbe interactions in postnatal life and the corresponding influences of gut microbes on early neurodevelopment may yield insights about the developmental origins of mammalian brain function and behavior in adulthood.

## Results

### Neonatal treatment results in stable colonization of the mouse intestinal tract by postnatal day 4

**Figure 1:**
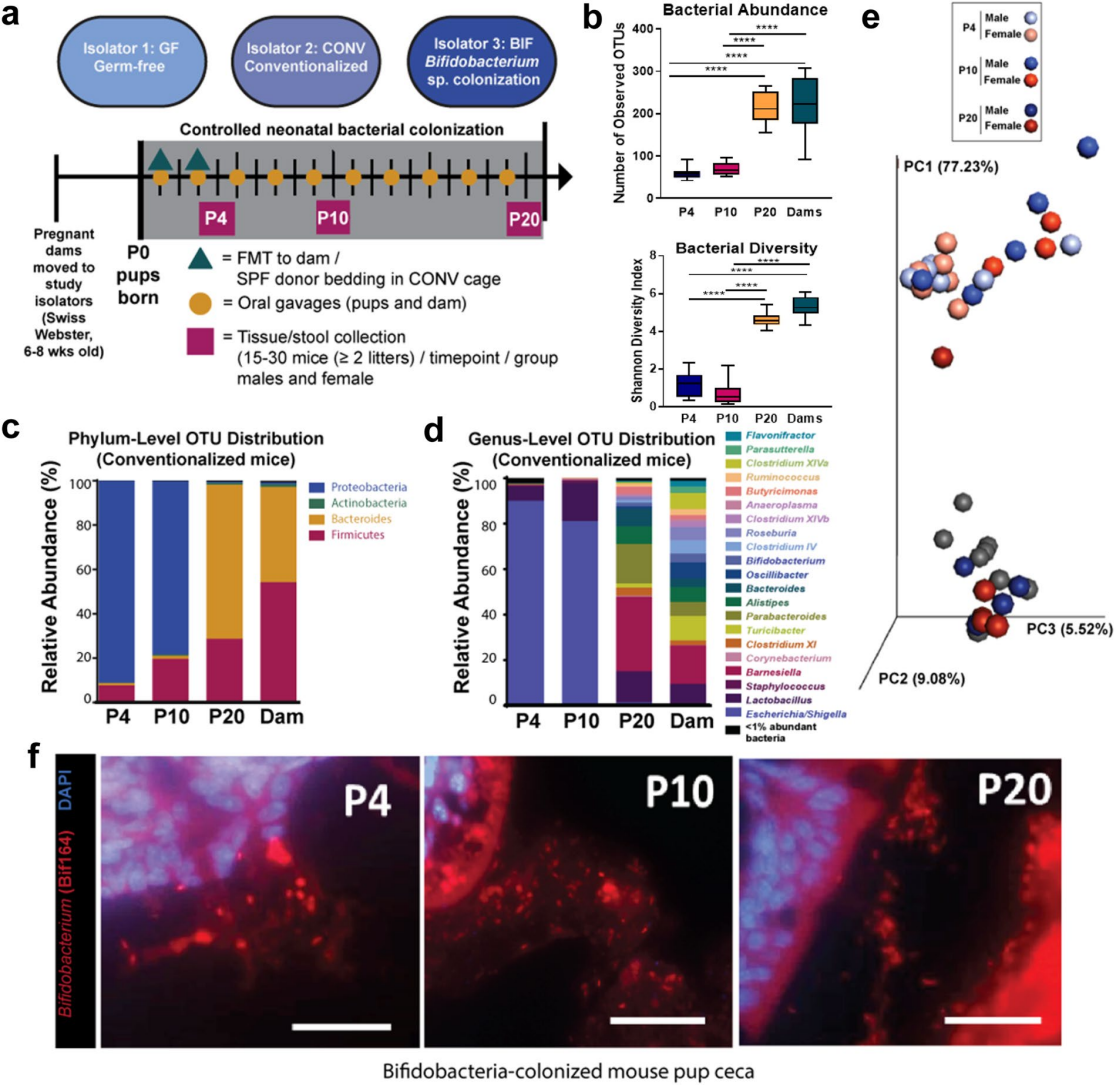
Neonatal conventionalized model replicates seeding conditions of intestinal microbiota by dam and environment (**a**) Timeline of controlled colonization of pups and dams in each gnotobiotic isolator occurred during the neonatal (P1-P21) developmental window. Pups received oral gavages of either the *Bifidobacterium* treatment (BIF) or sterile PBS (CONV and GF groups). The fecal slurries from SPF mice was delivered to the CONV dams on P1. See the Methods section for additional experimental detail. (GF = germ-free, CONV= Conventionalized, BIF=*Bifidobacterium*-colonized, P = postnatal day) (b-e) Data shown was generated from both male and females, n = 15-30 mice (>2 litters)/group/timepoint) (**b**) Alpha diversity of microbiota in each treatment group as measured by the Shannon Diversity Index (right panel) and Operational Taxonomic Unit (OTU) richness as measured by number of observed OTUs (left panel) (c-d) Longitudinal relative abundance of operational taxonomic units (OTUs). (**c**) Phylum–level comparison and (**d**) genus-level comparisons of OTU relative abundance in the CONV group of mice at each developmental timepoint. (**e**) Principal Coordinates Analysis demonstrating similarity between male and female fecal microbiome profiles in the conventionalized cohort. The percentage variation explained by each of the three primary principal factors is indicated on each axis. Coordinates representing individual samples are colored according to group, with distances to other coordinates indicating relative similarity/dissimilarity. (**f**) Representative micrographs (40x magnification) from Carnoy’s-fixed and paraffin-embedded cecal tissue from BIF mice at each postnatal timepoint. Slides were hybridized with the Texas Red-labeled *Bifidobacterium* genus-specific probe Bif164, demonstrating sparse colonization at early neonatal stages but increased colonization by P20. Nuclei of mouse intestinal epithelial cells are counterstained using DAPI.

Our model system of the infant microbiota consisted of neonatal gnotobiotic mouse pups colonized with four species of *Bifidobacterium* known to colonize the intestines of human infants in high abundance. As shown in the experimental timeline in Fig. 1a, the controlled colonization of pups and dams in each gnotobiotic isolator occurred during the neonatal developmental window starting at P1. The pups tolerated the modified gavage well, and no apparent injury or mortality was associated with the procedure. The germ-free control pups (GF) were treated with sterile saline, and the absence of microbial colonization was confirmed via routine agar plating of fecal matter throughout the experiment. Germ-free status was also confirmed in each cage of mice at sacrifice via agar plating of intestinal contents and fluorescence *in situ* hybridization (FISH) of intestinal tissue samples with universal bacterial probes.

Colonization of the conventionalized pups occurred in a very short time frame via exposure to used bedding (from a cage of SPF donor mice) added to the cage environment on days P1 and P3. An additional, and likely significant, source of microbial exposure was the dam, which was conventionalized via fecal microbiota transplantation (FMT) on days P1 and P3. This modified method of neonatal conventionalization (indirect colonization of pups via the dam and environment) was successful and resulted in colonization of pups with a complex microbiota by P4 (Fig. 1b–e). Additionally, as demonstrated by FISH staining, the early and consistent gavages of bifidobacteria resulted in stable colonization by P4 (Fig. 1f). The images demonstrate relatively sparse colonization at the earliest time point, but by P20, a dense community of bifidobacteria was established in the mucus layer above the epithelium.

As shown in Fig. 1b, bacterial abundance (top panel) and diversity (bottom panel) significantly differ at the first two neonatal time points, (P4 and P10) relative to the P20 mice and the adult dams. This pattern likely reflects changes in diet, as these mice begin to eat solid food around this time and are usually weaned from the dam’s milk by P21. We speculate that introduction of dietary fiber in solid foods promotes the colonization of Bacteroidetes, as demonstrated by other studies^34^. The genera that dominate the P4 and P10 conventionalized mouse gut are *Escherichia*/*Shigella* (phylum Proteobacteria) which represent approximately 80-90% of the neonatal gut microbiome during this time frame (Fig. 1c,d). This distribution is in stark contrast to the diversity and abundance of species observed in the P20 pups and the dams. Between P10 and P20, the microbial community shifts dramatically in composition, and by P20 the community is dominated by *Parabacteroides*, *Turicibacter*, and *Barnesiella* spp. Previous studies have established that conventionalized mice have bacterial communities which mirror the donor microbiome profile^35^. We observed a similar result in the P20 group of conventionalized mice in this study, whose microbial communities were closely aligned with that of the dam (the donor in this instance (Fig. 1d)). This finding further demonstrates the profound effect that diet has on the intestinal microbiome in early life: increased diversity and richness is expected to correspond with an increasingly complex set of microbial signals that change over time as the microbiome composition shifts and yields corresponding responses in the host CNS.

We have previously shown that the sex of an animal has a significant impact on microbiome composition in conventionalized mice, and that sex influences certain aspects of the microbiome-host interaction, affecting downstream behavior patterns as the mice age into adulthood^24^. In the postnatal mice (P4, P10, P20) in this study, there were no significant differences in microbial community profiles due to sex. The microbiomes of male and female conventionalized mice at each time-point were more similar to each other, than to microbiomes of mice of the same sex at different time points **(**Fig. 1e**)**. These data indicate that postnatal age, rather than sex, accounted for most of the microbial community variation (more than 77%) between groups of conventionalized mice in this study.

### Gene expression is modulated during postnatal development by microbial colonization

We assessed developmental expression of genes controlling synapse development and plasticity in the brain using a PCR array (Qiagen Mouse Synaptic Plasticity RT² Profiler PCR Array; all genes assessed in this array are listed in Supplementary Table S1). Synaptic plasticity is prominent during the period of postnatal neurodevelopment and is critical for ensuring functional neural circuitry^36^. This suite of genes was also an attractive target for our investigation as previous studies in adult rodents suggest that this set of genes and pathways are modulated by the intestinal microbiota^1,25^. We assessed expression of synaptic plasticity-related genes in the cerebellum, hippocampus, and cortex (isocortex, dorsal pallium) and compared gene expression within each brain region across treatment groups at each postnatal timepoint to detect microbial-driven fluctuations in gene expression during early life development.

**Figure 2:**
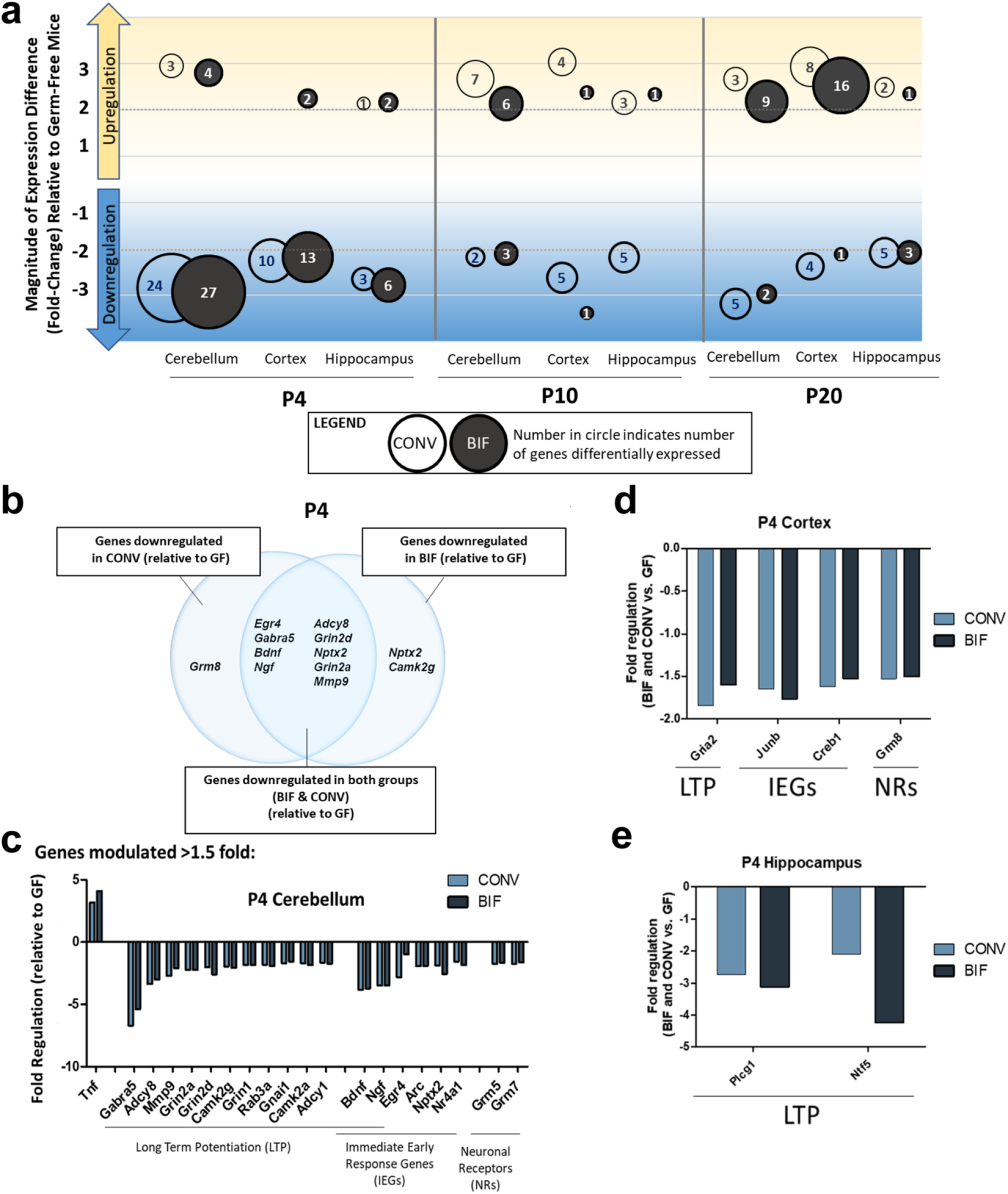
Summary analysis indicating significant downregulation of gene expression in colonized mice during early postnatal development. (**a**) Plot summarizes the results of the qPCR array. Values inside the circles represent the number of genes differentially expressed relative to germ-free control mice at each postnatal timepoint, in each brain region. The magnitude of the fold-change is indicated by position on the graph. CONV (open circle) and BIF (closed circle). (**b**) Comparison of downregulated genes in P4 mice (BIF relative to GF, CONV relative to GF, and genes downregulated in both BIF and CONV mice relative to GF). (**c**-**e**) Genes differentially expressed in BIF and CONV mice >1.5 fold in the P4 mice Cerebellum (**c**), Cortex (**d**), and Hippocampus (Figure 1.) (*No error bars or statistical significance bars are shown due to the pooled samples used for this analysis, PCR array of RNA from n = 5 male mice/group/timepoint/brain region).

Biologically significant differences in gene expression between treatment groups were observed at P4 in all 3 brain regions. This observation suggests that synapse-related genes are upregulated in germ-free animals early in life relative to mice colonized with gut microbes (Fig. 2a). As the RNA samples used in this analysis were pooled (from n = 5 male mice/group/timepoint/brain region), the results shown in Fig. 2 do not show error bars or statistical significance, rather they are shown to illustrate the trend in expression differences observed, and to highlight the genes that were similarly up/down regulated in BIF and CONV mice relative to GF. (See Supplemental Table S2 for full list of genes that were differentially regulated >1.5 fold at all timepoints/brain regions). Genes which were observed to be up- or down-regulated more than 1.5-fold in the CONV and BIF groups relative to GF mice at the P4 time-point are compared in Fig. 2b and further detailed in Fig. 2c–e. The expression pattern observed indicates that gene expression at the earliest postnatal timepoint may be most sensitive to microbial colonization. The synapse-related gene expression changes observed in the cerebellum and hippocampus also concur with results of studies previously performed in adult rodents^1^. In mice, cerebellar development continues well after birth, which may make the region more susceptible to environmental influences, such as the influence of microbial signals originating during the period of microbial colonization after birth^32,37^. Conversely, brain regions that develop prenatally are thought to have more limited exposures to microbes and microbial metabolites *in utero*^38^.

Many genes that were differentially regulated in the CONV groups were similarly regulated in the BIF groups, indicating that the host responses are predictable. This leads to the possibility that a defined pattern of host CNS gene expression may be generated by colonization with defined subsets of gut microbial communities. At P4, genes and gene pathways that play a vital role in synapse formation in the developing brain were similarly down-regulated in both groups of mice colonized by gut microbes (CONV and BIF) relative to germ-free mice (Fig. 2b**)**. In the P4 cerebellum, many genes involved in long term potentiation (LTP), as well as immediate early response genes, which can also play a role in LTP, were down-regulated in colonized mice (Fig. 2c). Both gene pathways play a vital role in activity-dependent synapse formation in the developing brain. This pattern holds true in other brain regions as well, with genes involved in LTP being downregulated in the hippocampus and cortex in both colonized groups of mice, although the number of downregulated genes was fewer (Fig. 2d-e). In follow-up experiments using adult gnotobiotic mice mono-colonized with *Bifidobacterium* (*B. dentium)*, we observed a similar effect. Synaptic genes (such as *Dlg4)* were also significantly downregulated in bifidobacteria-treated adult mice relative to germ-free adult mice (Supplemental Figure S1 and Supplemental Table S3).

### Neonatal colonization with *Bifiodbacterium* influences microglia phenotypes

Microglial-dependent synapse elimination also represents a potential microbiota-driven mechanism that influences neural circuit maturation. Microglia are key mediators of synapse remodeling during postnatal development^32,39–43^ and serve to eliminate supernumerary and inappropriate synapses, resulting in the functional maturation of the remaining appropriate synapses. Microglia-mediated phagocytosis is a mechanism that helps ensure network refinement and functional organization of circuitry^40,44–46^. We examined cell surface markers, TNF expression, and morphology in our analysis in order to characterize the microglial populations present in our neurodevelopmental mouse model.

**Figure 3:**
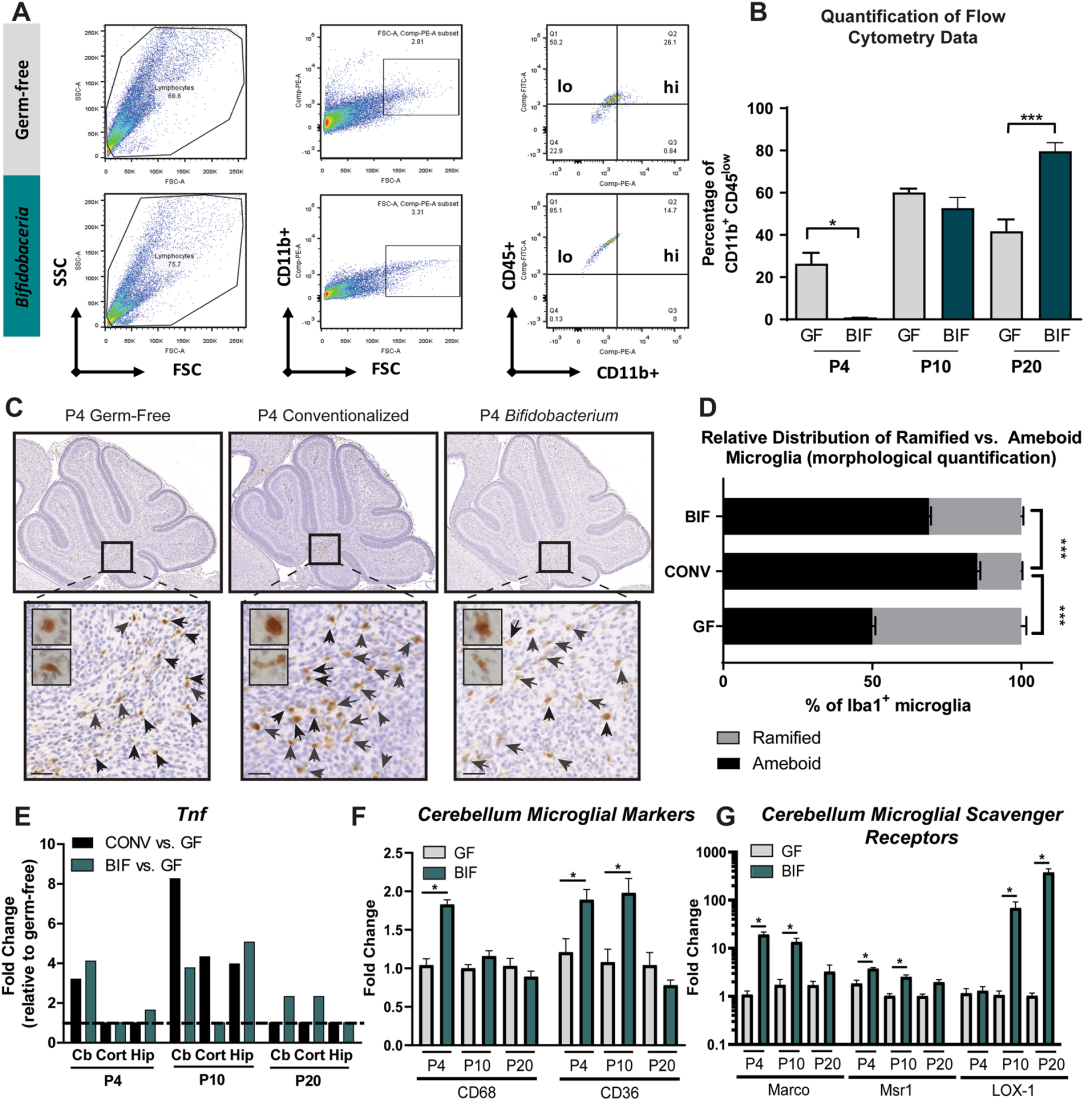
Colonization with conventional microbiota and bifidobacteria facilitates early microglial reactivity and mid-late microglial ramification (**a**) Representative image of gating strategy for flow cytometric analysis. (**b**) Results plotted as percentage of CD11b^+^ CD45^low^ microglia/total microglia at each timepoint. Ramified microglia possess the phenotype CD11b^+^,CD45^low^. At P4, GF mice have an increased % of microglia expressing markers of ramified microglia, relative to BIF mice. (One-Way ANOVA; *p<0.05, **p<0.01, *** *p*<0.001; n = 3 male animals/group/timepoint) (**c**) Microglia were labeled using an anti-IBA1 (Ionized calcium binding adaptor molecule 1) antibody. Scale = 50 μm. Representative images of staining in cerebellar white matter of P4 pups in each group, demonstrating fewer ameboid microglia in P4 germ-free mice (**d**) Morphological classification and quantification of ramified vs. ameboid microglia in P4 mice (**** *p*<0.0001, n = 3 mice/group, with 3 fields per mouse). (**e**) Expression of *Tnf* gene from whole tissue homogenate in each region (Cb = Cerebellum, Cort = frontal cortex, Hipp = Hippocampus) (data generated from PCR array data, therefore statistical significance and error bars are not shown for these pooled samples; n = 5 male mice/group/timepoint/brain region) (**f**) qPCR analysis of GF and BIF mouse cerebellum for markers of phagocytic microglia. (One-Way ANOVA; *p<0.05; n = 8 male animals/group/timepoint) (**g**) qPCR analysis of GF and BIF mouse cerebellum for scavenger receptors, which are required for proper phagocytosis. (One-Way ANOVA; *p<0.05; n = 8 male animals/group/timepoint).

During normal development, phagocytic (ameboid) microglia are abundant early in life, and actively participate in synaptic remodeling^37,39–41^. Ameboid microglia participate in synaptic pruning and phagocytosis during the postnatal period to help refine the network as it develops by removing superfluous axons and promoting axonal migration and growth^37,40,41,47–49^. Over time, the microglia enter a ramified state, in which they are surveilling, but not phagocytosing (unless activated in response to injury or pathogen invasion)^40,50,51^. Flow cytometry is traditionally used to characterize the cell surface markers, or antigenic expression, of microglia in order to differentiate between phenotypes^52,53^. Ramified, surveilling microglia possess the phenotype CD11b^+^ and CD45^low^ whereas microglial precursors, CNS-infiltrating macrophages, or activated microglia exhibit CD11b^+^, CD45^high^ phenotype^42,53–55^. During a critical window of normal neurodevelopment (between P2 and P14), the population of ramified microglia expressing CD11b^+^, CD45^low^ expands^56–58^. By P20 the adult population of microglia is well established and mostly in a ramified state under normal circumstances^59^. We used flow cytometry to assess expression of these surface markers indicative of ramified microglia and examined the average percentage of CD11b^+^, CD45^low^ expressing cells between groups (Fig. 3a,b). At the P4 time point, the populations of surveilling microglia are low in both the GF and BIF groups of mice, though we observed significantly fewer cells expressing CD11b^+^, CD45^low^ in the BIF group relative to the GF group. The population of CD11b^+^, CD45^low^ expressing cells rapidly expanded between P4 and P10 in the BIF mice when compared to a smaller expansion observed in the GF mice. By P20, when the adult population of ramified microglia should be well established, approximately 80% of the CD11b^+^ cell population is CD45^low^ in the BIF group, compared to only approximately 40% in the GF group. This rapid expansion of ramified microglia between P10 and P20 is expected in a healthy mouse colonized with a complex gut microbiome^32^. Our results indicate that a simplified, bifidobacteria-predominant gut lumen at these postnatal timepoints also elicits the expansion of ramified microglia in the brain. These data reveal a delay in the development of the immune system in the GF group, which is a well-documented phenomena typically observed in GF animals^60–62^. Due to the dual role of microglial cells, this early deficit, even if only a delay, may have a large impact on the formation of neural circuitry and elicit downstream effects on brain function later in life.

During development (from P2-P14 in mice), microglial morphology also follows a general pattern, from amoeboid or reactive cells, to ramified or surveilling microglia^37,56,63–65^. The ameboid-like morphology, characterized by large rounded cell bodies and lack of processes, is generally considered functionally active, phagocytic-type, which are observed in large numbers early in development^37,65^. The ratio of ramified:ameboid cells increases at late postnatal timepoints, with ramified cells being characterized by a flattened morphology with many complex process arbors^37^. We therefore utilized cellular morphology to further assess microglial reactivity. Microglia were specifically immunolabeled using an anti-IBA1 antibody (Ionized calcium binding adaptor molecule 1) to examine their cellular morphology^66–70^. Figure 3c shows representative images from P4 mice in each treatment group, and the insets demonstrate the staining and differences in ameboid vs. ramified morphology. Figure 3d quantifies the relative distribution of ramified:ameboid microglia at the P4 time point in each group of mice. At this early P4 time point, the CONV and BIF animals have a dominant percentage of microglia classified as ameboid, indicating phagocytic activity. In contrast, GF animals had fewer ameboid microglia relative to BIF and CONV mice. These data further indicate that microbial colonization can influence microglia reactivity.

After exposure to environmental or microbial signals, microglia in an activated state will release and/or upregulate production of various pro- and anti-inflammatory mediators such as TNF-α^71–73^. In the brain, TNF-α is primarily synthesized by reactive microglia^72,74,75^. Microglial TNF-α released near synapses can participate in the functional maturation of inhibitory and excitatory circuits by increasing neuronal expression of neurotransmitter receptors^76–78^. Abnormal levels of TNF-α have been linked to reduced complexity in cultured neurons and premature stabilization resulting in insufficient refinement during development *in vivo*^79–81^. Upregulation of the *Tnf* gene in these brain regions at the early postnatal time points (P4 and P10) in the BIF and CONV groups of mice relative to the GF mice is therefore an additional indicator of early microglial activity in the colonized mice (Fig. 3e**)**. In addition to cytokine production, another important function of microglia is phagocytosis. We examined *CD68*, a transmembrane glycoprotein that indicates phagocytic activity^82^, and *CD36*, a pattern recognition receptor involved in phagocytosis^83^, by qPCR in the cerebellum of GF and BIF-treated mice at P4, P10, and P20 (Fig. 3f). We observed increased expression of *CD68* and *CD36* at P4 and increased *CD36* at P10 in BIF mice compared to GF. Interestingly, these markers were not differentially expressed at P20 in both GF and BIF mice, which suggests the importance of early neuronal immune modulation. We also examined scavenger receptors, which promote phagocytosis and clearance by microglia^84^, via qPCR in the cerebellum of GF and BIF-treated mice at P4, P10, and P20 (Fig. 3g). We found increased expression of *Macro* and *Msr1* during early development (P4 and P10) and increased expression of LOX-1 receptors at P10 and P20 in BIF mice compared to GF. These findings further point to potential functional differences in the activity of microglia in colonized mice versus those lacking an intestinal microbiome.

### Neonatal colonization modulates synaptic density and neural circuit maturation

Overexpression of synapse-related genes may serve to strengthen activity-dependent synapse formation in the brains of germ-free mice, leading to an aberrant increase in synapse formation. The lack of synaptic pruning due to lack of reactive functional microglia in germ-free mice may also contribute to the observed increase in synaptic density. In germ-free mice, the concurrent upregulation of synaptic genes and lack of reactive, functional microglia early in postnatal neurodevelopment may result in a system of ineffective pruning resulting in potentially dysfunctional or non-functional synaptic connections, thereby affecting the development of matured and functional neural networks. We chose to use the well-described cerebellar circuit^85–88^ as a model to examine how the above described effects may influence the development of functional neural circuits.

**Figure 4:**
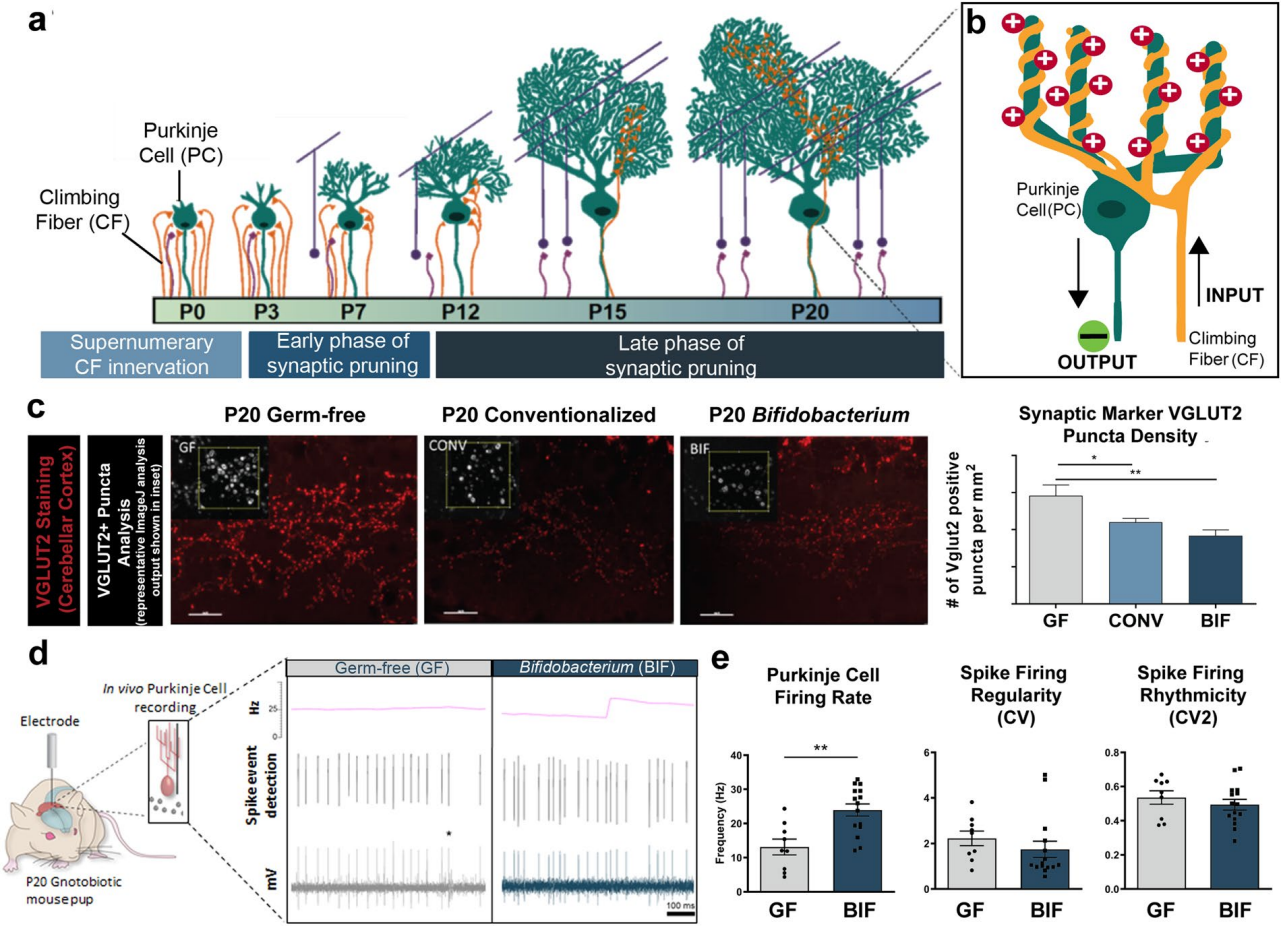
Germ-free mice display aberrant synaptic density and synaptic function that is normalized by bifidobacteria colonization (**a**) Simplified diagram of the cerebellar circuit over postnatal development and corresponding phases of synaptic refinement. (**b**) Inset illustrates interaction between two cerebellar cell types: Purkinje cells (PC; main functional output of cerebellum) and climbing fibers (CF). CF synapses on PCs are initially supernumerary, but eventually reach a 1 CF: 1 PC ratio with 1 CF terminals synapsing on multiple locations of PC dendrites. The circuit diagram also shows simplified signaling input and output between the cell types in this model system. (**c**). Micrographs (40X magnification) of molecular layer of cerebellum in lobule II of brain slices from P20 male mice in each group stained with anti-VGLUT2 antibody. Puncta of the pre-synaptic marker VGLUT2 were counted in a 400 micron field. Inset demonstrates puncta analysis in ImageJ. The density of the pre-synaptic marker VGLUT2 per field is quantified in the right panel as VGLUT^+^ puncta per mm^2^ *p<0.05, **p<0.01, One-way ANOVA (3 fields per section with 3 sections per animal, *n* = 3 animals per group) (d) Schematic of *in vivo* electrophysiological recording of Purkinje cells in the cerebellum, and representative extracellular single-unit recording traces from male germ-free and bifidobacteria-colonized mice. Asterisk indicates low-frequency complex spikes that are triggered by climbing fiber input, which identifies the cell as a Purkinje cell. The instantaneous firing rate is indicated by the pink line above the raw traces. (Figure 1). Firing characteristics of Purkinje cells quantified from *in vivo* electrophysiological recordings of GF and BIF anesthetized P20 male mice (*n* = 3-5 animals/group with 3 separate recordings per animal).

The diagram in Fig. 4a demonstrates a simplified summary of interactions between two cerebellar cell types (Purkinje cells in green and climbing fibers in orange) during development and shows how these interactions change over time. At P0 many climbing fibers contact a single Purkinje cell and have supernumerary synaptic contacts. Normally, climbing fiber synapses are pruned until only one fiber innervates a single Purkinje cell by the late phase of synaptic pruning at ~P20^89–92^ (Fig. 4b). We predicted that these contacts would remain supernumerary in GF mice due to their upregulated synapse-related gene expression and dampened microglial response. We assessed synaptic density changes by examining the climbing fiber to Purkinje cell contacts over postnatal development in GF, CONV, and BIF mice. VGLUT2 immunostaining labels climbing fiber pre-synaptic terminals in the molecular layer of the cerebellar cortex and therefore serves as a proxy for examining the pruning of these supernumerary climbing fiber synaptic connections with Purkinje cells during postnatal development.^93–95^. Fluorescence immunohistochemistry was used to visualize the synaptic marker VGLUT2 and the Puncta Analyzer plugin in ImageJ was used to count the number VGLUT2-labeled puncta per 400 µm^2^ field. Representative images of this staining and the ImageJ puncta analysis are shown in Fig. 4c. The results are quantified as number of VGLUT2 positive puncta/mm^2^ and demonstrate a significant increase in the number of puncta in germ-free mice at P20 (Fig. 4c**)**. This increased number of puncta corresponds to an increase in the pre-synaptic marker VGLUT2 in the GF group relative to both the CONV group and the BIF group at P20. The increased number of the pre-synaptic marker in germ-free mice was normalized by postnatal conventionalization and *Bifidobacterium* colonization.

Additionally, *in vivo* electrophysiology was conducted in anesthetized mice in order to measure neuronal activity using the same model circuit formed between Purkinje cells and their climbing fiber inputs^96^). We obtained extracellular single-unit electrophysiological recordings from Purkinje cells as previously described (Fig. 4d)^97^. Purkinje cells in germ-free mice were observed to have a significant decrease in Purkinje cell firing rate relative to *Bifidobacterium*-colonized mice at P20 (Fig. 4e). Other recorded measures of cell activity (spike firing regularity and rhythmicity) did not yield differences. These findings further support the hypothesis that the lack of microglial function and increased synaptic density in the cerebellum creates a dysfunctional circuit in germ-free mice. Although not specifically addressed in this study, it would reason that other regions of the brain are similarly affected in early life by this or a similar mechanism, which may explain the abnormal germ-free mouse behaviors previously documented.

## Discussion

This work is the first demonstration that specific synaptic genes and functional neural circuitry are modulated during development of the mammalian CNS in response to a group of bacteria residing in the gut. Our work demonstrates that the CNS of germ-free mice are characterized by upregulated synapse-promoting genes and a relative lack of microglial reactivity early in postnatal development. These results are consistent with reports that microbial signals promote microglial development^32^, which ultimately refine the network and ensures functional organization of neural circuitry. This combination leads to supernumerary synaptic inputs which are unable to be effectively refined by synaptic pruning or phagocytosis. We are the first to show that postnatal microbial colonization modulates gene expression in the CNS, downregulating certain synapse-promoting genes during the early postnatal time period. This study demonstrates that neonatal colonization with a complex adult murine microbiota also promotes microglial reactivity and affects synaptic density, thereby affecting neuronal circuit function. Moreover, our work demonstrates that a complex microbiota was not necessary to elicit these benefits, as postnatal colonization with only four key *Bifidobacterium* species yielded similar effects on neurodevelopment. This work further supports the importance of *Bifidobacterium* as members of the healthy infant gut microbiota and highlights their potential roles in neuromodulation.

**Figure 5:**
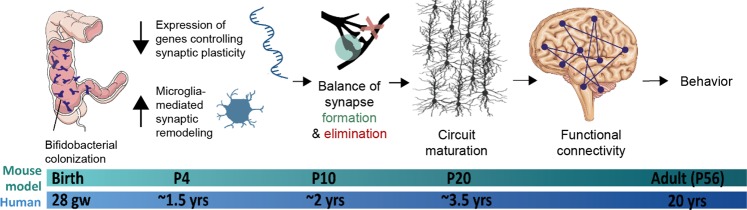
Proposed mechanism of bifidobacteria-mediated effects on host neurodevelopmental processes. In this proposed model, bifidobacteria down-regulate early expression of synapse-related genes in the host CNS. Additionally, bifidobacteria are important for the early reactive functionality of microglia, which prevent excess synapse formation during this critical window. This process promotes effective refinement of the developing synaptic network and potentiation of appropriate contacts, leading to circuit maturation. Functional organization of neural circuitry leads to synchronicity of firing and typical behavioral phenotypes.

This work is among the first to demonstrate that germ-free mice exhibit an early upregulation of synapse-promoting genes, which can be down-regulated by both neonatal conventionalization and *Bifidobacterium*-colonization. Additionally, the microglia in CONV and BIF mice are in a reactive state early in development, whereas GF mice exhibit impaired microglial function. This upregulation of synapse-promoting genes and a lack of microglial reactivity in germ-free mice during early postnatal development leads to supernumerary synaptic inputs that are not effectively refined by synaptic pruning or phagocytosis. By the P20 time point, germ-free animals have increased synaptic density corresponding with decreased neuronal function relative to colonized mice (either bifidobacteria-treated or conventionalized). Overall, we propose that in GF animals, synaptic changes resulted in circuit connectivity defects, leading to the aberrant behavioral phenotypes observed in adult germ-free mice. However, these effects can be prevented if mice are postnatally colonized, even by a limited consortium of key *Bifidobacterium* species (summarized in Fig. 5).

Gene expression profiling at each postnatal time point using the RT² Profiler PCR Array system (Qiagen) served as a sensitive tool for analyzing the expression of many pathway-focused genes simultaneously. Physical and gene expression changes at neuronal synapses are characteristic of synaptic plasticity. Expression of immediate-early genes (IEGs) is altered immediately after neuronal events characteristic of synaptic plasticity. LTP is a process that strengthens synaptic connections, and it is mediated by upregulation of IEGs. Co-upregulation of certain genes in germ-free mice indicates IEG and LTP pathway co-upregulation. For example, *Bdnf* expression requires prior induction of the IEG *Arc*. Both *Bdnf* and *Arc* are increased in the germ-free cohort indicating pathway-focused upregulation. Many of the genes regulated by microbial colonization are those involved in LTP as well as other IEGs which play a role in LTP. In addition, many essential neuronal receptors are expressed in the postsynaptic density, a specialized segment of the neuronal membrane. We observed that postnatal microbial colonization of the gut with both a complex microbiota and the simplified 4-member microbiota (*bifidobacteria*), resulted in the down-regulation of many genes related to these processes, ultimately serving to prevent the formation of excess synapses. Our data describing microbiota-mediated effects on microglia also concur with recent literature, and demonstrate that microbial signaling significantly impacts microglial reactivity^32,43,98,99^. Future studies of how the microbiota influences microglia should confirm changes in microglial function using additional markers of maturation. We show that deficient synaptic pruning is associated with altered synaptic transmission and decreased functional brain connectivity, which may be an underlying mechanism contributing to the behavioral deficits in sociability^24^ and repetitive-behaviors observed in many previous studies of germ-free animals. This is a unique finding as the link between weak transmission in the cerebellum and social behavior is understudied in repetitive behaviors.

Consistent with published literature, our work also demonstrates that immediately after birth, the neonatal gut is easily dominated by facultative anaerobes, like the genus *Escherichia*. These facultative anaerobes that colonize the infant gut act as “pioneers”, and by consuming oxygen and altering the pH, they make the environment better suited and amenable to strictly anaerobic bacteria like *Bacteroides* and *Bifidobacterium*^100^. This pattern is faithfully replicated in our study, in which P4 and P10 CONV mice are heavily colonized by microbes of the genus *Escherichia* right after birth, but during the subsequent months, the gut is gradually colonized by increasing numbers of strict anaerobes like *Bacteroides*. Our model of colonization appears to be similar to the natural route of colonization (from the environment and the dam rather than from the gavage) and also replicates the expected succession of bacterial species in the intestine. We hope that this model may serve future researchers in examining further attributes of the gut-brain axis during development.

Currently, there is limited information regarding the exact mechanisms by which gut microbes communicate with the host brain. Proposed mechanisms of microbiome-gut-brain communication including immune system activation, signaling via vagal afferents, and production of microbial metabolites that can either directly or indirectly affect the brain. This study points to a mechanism that can be activated by both CONV and BIF groups of mice. SCFAs have been linked in previous studies to microglial maturation^32^ which influence synapse elimination^101^. Acetate, in particular, has also been linked to epigenetic modifications in the brain that affect gene expression^102^. Mice deficient for the SCFA receptor FFAR2 mirrored microglia defects found under GF conditions^38,103^. *Bifidobacterium* species produce large quantities of acetate, which may explain their ability to mediate effects similar to that of a complex microbiota. Likewise, immune-mediated effects would be present in both groups and could also be the causative mechanism underlying the phenomenon described herein. Finally, bacterial metabolites may directly affect neuronal morphology activity, independent of the immune system; an effect that has been observed in *C. elegans*^104^. Future work in the field should focus not only on specific microbial metabolites, but should take a systems-based approach in determining how specific bacterial communities elicit their effects on the host CNS.

Aberrant expression of genes involved in neuronal signal transmission and synaptic plasticity are implicated in neurodevelopmental disorders and there are many non-genetic (environmental) factors that are known to affect expression of these genes^105^. Disruption of the intestinal microbial ecosystem (dysbiosis) at an early age may play a role in neurodevelopmental disorders, such as autism spectrum disorder. Due to limitations based on our study design, we did not perform correlation analyses between the microbiota and gene expression, though future studies should address this in order to determine causality. We currently have limited information regarding how early colonization with *Bifidobacterium* affects neurodevelopment, however there are several neuropsychological and neurodevelopmental disorders that are associated with a decreased abundance of bifidobacteria. While this correlation does not demonstrate causation, the mechanisms described herein may play a role in the etiology of some of these pathophysiologies. With enhanced knowledge of how this beneficial genus affects the CNS in early life, novel therapeutic strategies may be designed to exploit these species in order to elicit desired effects on the brain. The work presented herein offers a more complete description of how the postnatal colonization of the intestine by *Bifidobacterium* spp. influences plasticity in the developing brain. By examining how these species are able to affect brain development, we may be able to identify and/or engineer specific bacterial communities to be maximally beneficial for human neurodevelopment, including the functional enhancement of the microbiome:gut:brain axis.

## Methods

### Bacterial strains, media, and culture conditions

The following bacterial strains were obtained from the American Type Culture Collection (ATCC): *Bifidobacterium dentium* (ATCC 27678), *Bifidobacterium longum* subspecies *infantis* (ATCC 15697), *Bifidobacterium breve* (ATCC 15698), and *Bifidobacterium bifidum* (ATCC 29521). These strains were cultured in anaerobic de Man, Rogosa and Sharpe (MRS) medium (BD, Sparks, MD, USA). Cultures on agar plates and liquid cultures were grown under anaerobic conditions (Anaerobe Systems, Morgan Hill, CA) at 37 °C. Additional information listed in Table 1.

**Table 1:** *Bifidobacterium* species used to examine effects of neonatal colonization on mammalian neurodevelopment.

Species/Strain designations	BSL	Relevant features	Growth Conditions	Source
*Bifidobacterium longum* subsp. *infantis* ATCC 15697 Strain: S12	1	-Isolate from human infant intestine -Genome assembled and annotated	Anaerobic, MRS plates and liquid medium, 37°C	ATCC
*Bifidobacterium bifidum* ATCC 29521 Strain: VPI 11241 [Ti]	1	-Isolate from human infant feces -Genome assembled and annotated	Anaerobic, MRS plates and liquid medium, 37°C	ATCC
*Bifidobacterium dentium* ATCC 27678 Strain: B757	1	-Isolate from human infant feces -Genome assembled and annotated	Anaerobic, MRS plates and liquid medium, 37°C	ATCC
*Bifidobacterium breve* ATCC 15698 Strain: S50 variant a	1	-Isolate from human infant intestine -Genome assembled and annotated	Anaerobic, MRS plates and liquid medium, 37°C	ATCC

BSL = Biosafety Level.

MRS = Man, Rogosa and Sharpe medium.

Anaerobic gas mix = 80% N_2_, 10% CO_2_, 10% H_2_.

ATCC = American Type Culture Collection.

### Germ-free mouse models

Germ-free (GF) Swiss Webster mice (Taconic) were bred in a germ-free facility at Baylor College of Medicine. The outbred colony of germ-free mice was maintained in a germ-free “colony isolator”. Swiss Webster mice are attractive as an outbred mouse model due to large litter sizes, nurturing instincts, and previous microbiome-gut-brain studies, making this strain useful for cross-study comparisons. Timed pregnancies were induced in adult female mice at approximately 6-8 weeks of age, in the germ-free colony isolators. Vaginal plugs were used to identify pregnant dams and time the pregnancy. Pregnant germ-free females were randomly assigned to an experimental group, and moved (under germ-free conditions) to the respective study isolator before the day of birth, which was considered as P0. Pregnant dams and their litters were maintained in separate cages within these 3 identical, but separate, flexible isolators fed with HEPA-filtered air. Each isolator housed only the dams and litters within a single experimental group in this study. Mice in each isolator were maintained under germ-free conditions or colonized with bacteria and maintained in “gnotobiotic conditions”. Offspring in each treatment group raised in these germ-free and gnotobiotic isolators were handled in the same manner. Dams and their respective litters in were kept together in filter-topped cages in the isolators pre-weaning, and in groups separated by sex, in filter-topped cages in the same isolators after weaning. All animals had access to irradiated food and water *ad libitum*. All animals were housed under 12 h light/dark cycle. Animal care and experimental procedures were approved by the Institutional Animal Care and Use Committee (IACUC) at Baylor College of Medicine, Houston, TX, in accordance with all guidelines set forth by the U.S. National Institutes of Health. Germ-free control mice received gavages of sterile 0.1 M phosphate buffered saline (PBS; Gibco) in the same manner as the experimental mice (described in section below). Germ-free status was monitored over the course of the experiment by plating fresh fecal pellets from the dams in each cage within the germ-free isolator on blood agar plates (Hardy Diagnostics) and incubating aerobically and anaerobically at 37 °C.

### Colonization of germ-free mice with bifidobacteria

Bifidobacteria were grown in culture conditions described above. Bacterial cultures grown overnight (approximately 12 h) were harvested in log phase. Cultures were combined in equal ratios based on OD_600_ readings (volume equal to approximately OD_600_ 1.0 for each of the species; approximately 10^9^ CFU), pelleted, then re-suspended in 3 mL sterile PBS. Colonization of males and females with bifidobacteria was achieved by administering 0.2 mL of this treatment directly to the stomach of each dam via oral gavage on alternating days post-partum. The treatment was also administered with the drinking water. Pups were administered 0.02 mL of the treatment mixture starting at P1, and on alternating days until P20. For pups between the ages of P1-P10, we administered the mixture orally via pipette tip. From P10 onwards, the oral gavage treatment was administered with an 18-gauge ball-tipped gavage needle. This persistent treatment ensured that all four human *Bifidobacterium* species were present during the postnatal window of interest in this study, irrespective of their ability to colonize the murine intestine in the absence of human milk oligosaccharides. Dilution plating of the treatment mixture on MRS plates at several timepoints during the experiment confirmed that the dams received approximately 1.1x10^10^ total CFUs (viable bacterial cells) per gavage. The pups received a smaller volume of the gavage mixture, approximate to 1.1x10^9^ total CFUs per treatment. Colonization was monitored over the course of the experiment by plating fecal samples from the dam and pups on MRS agar plates and incubating at 37°C anaerobically. Growth on plates after 48 h was considered positive verification of successful colonization. All dams and pups showed consistently positive colonization after the first 2 d of treatment.

### Colonization of germ-free mice with murine microbiota (conventionalization)

Conventionalized litters were colonized neonatally via indirect colonization of the dam and environment. Conventionalized males and females were derived by administering fecal slurries from specific pathogen free mice (SPF), which harbor a complex microbial community, via oral gavage to the dams on P1, and by adding used bedding (containing fecal pellets) to the cages on P1 and P3. A single cage of female, age-matched Swiss Webster mice were used as donors for each experimental cage and were the source of both the FMT and the dirty bedding transfer to avoid as much inter-individual variation as possible. The microbiome composition of the SPF mice used in this study as microbiome donors was examined in a previously published study^24^, which demonstrated that the taxonomic distribution of the conventionalized group of mice replicated the SPF donor fecal microbiome profile. Agar plating of intestinal contents from these pups on blood agar plates confirmed that this method effectively resulted in gut microbial colonization of the pups by P4. In order to maintain consistent handling throughout development between the groups, conventionalized pups (male and female) were also administered a sham gavage of sterile PBS in the same manner as described above. Colonization with conventional murine microbiota was monitored over the course of the experiment by plating fecal samples from the dam and pups on blood agar plates (Hardy Diagnostics) and incubating at 37 °C aerobically and anaerobically. Growth on plates after 48 h confirmed presence of complex microbiota consisting of multiple species and was used as positive verification of successful colonization.

### Adult mono-association with Bifidobacterium dentium

To colonize mice with a single *Bifidobacterium* strain, also known as mono-association, adult (7-9 month old) male and female germ free mice, *B. dentium* ATCC 27678 was grown in culture conditions as described above. Bacterial cultures were incubated anaerobically until administration to mice. Mice were administered approximately 3.2x10^8^ CFU of *B. dentium* per each oral gavage treatment. Control germ-free mice received sterile MRS broth. Oral gavages were administered once every other day for one week with one supplemental gavage one week later (4 total treatments). Colonization and germ-free status were monitored over the course of the experiment by plating fecal samples from the mice on MRS agar plates, and incubating at 37°C in aerobic and anaerobic conditions.

### Immunohistochemistry

For preparation of brain tissue for immunohistochemistry, vaporized isoflurane was administered at 1-4% with pressurized oxygen to maintain an appropriate plane of anesthesia during the perfusion. Once adequate depth of anesthesia was confirmed, blood was flushed from the circulatory system by a trans-cardiac perfusion with PBS. Then, the tissue was fixed by perfusing the animal with 10-50 mL of 4% paraformaldehyde (PFA) in PBS. The brain was then dissected and post-fixed for 24 h in 4% PFA. The brain was cryoprotected stepwise in 15% and 30% sucrose in PBS, embedded in Tissue-Tek® optimal cutting temperature compound (O.C.T., Sakura), and sectioned at 8 μm on a cryostat.

Immunohistochemistry was performed directly on electrostatically coated slides as described previously^106,107^. Briefly, sagittal sections were incubated in PBS containing 10% normal goat serum (NGS; Sigma Aldrich), 0.1% Tween-20, and primary antibodies for 16-18 h at room temperature. Microglia were labelled using rabbit anti-ionized calcium binding adaptor molecule 1 (IBA1, Wako, 1:400). Presynaptic elements on climbing fibers synapsing with Purkinje cells in the cerebellar cortex were labeled using mouse anti-vesicular glutamate transporter 2 (VGLUT2, Millipore, 1:500). The tissue sections were washed three times in PBS before incubation with secondary antibodies for a maximum of 2 h at room temperature. For chromogenic immunostaining of IBA1-labelled microglia, goat anti-rabbit horseradish peroxidase (HRP)-conjugated secondary antibody (Dako, 1:200) and the peroxidase substrate 3,3’-diaminobenzidine (DAB; Sigma-Aldrich) were used. Donkey anti-mouse Alexa-555 secondary antibody (Molecular Probes, 1:1500) was used for fluorescence immunostaining of VGLUT2-labeled puncta. The slides were washed and cover-slipped. (Imaging of slides described in section below).

### Analysis of immunostained tissue

A Zeiss Axioscan.Z1 microscope with an automated and motorized stage was used to capture micrographs of tissue sections. For analyses of microglial morphology, multiple images were collected from the same stained brain section and stitched together to produce a mosaic representing the entire section. Three mice from each group at each timepoint were analyzed. Stained cells with large, rounded cell bodies and lack of processes were scored as ameboid, and cells which had a flattened morphology with many processes were scored as ramified. Mosaic images were used for analysis of microglial morphology and counts of ameboid and ramified cells were performed manually (n = 3 mice/group, with 3 fields per mouse). For analysis of VGLUT2 puncta density, images were obtained using a 633 nm filter on a fluorescence microscope. Lobule II of the cerebellum in each section was located and Z-stack images at 40x magnification were taken. Synaptic density was measured in several 400 µm^2^ fields per image using the Puncta-Analyzer in Fiji-ImageJ (3 fields/ section with 3 sections/ animal; n = 3 male animals analyzed per group).

### rRNA sequencing and analysis

The gut microbial community was assessed using fecal and cecal samples from the dam and pups at each postnatal time point (male and female, n = 15-30 mice (>2 litters)/group/timepoint). All samples were collected under the sterile conditions and were stored at −80°C until processing. Bacterial DNA was extracted using the MoBio Powersoil DNA Extraction Kit (Qiagen). Texas Children’s Microbiome Center performed the amplification and sequencing of the Bacterial DNA as previously described^24^. Gene sequence libraries (targeting the V4 region of the 16S rRNA gene) were generated using the NEXTflex™ 16S V4 Amplicon-Seq Kit 2.0 (Bioo Scientific, Austin, TX). These libraries were sequenced on the Illumina MiSeq platform (median of 24,181 sequences/sample, mean of 25,801 sequences/sample). Host (mouse) DNA sequences were filtered from the sequence libraries using Bowtie2^108^. Sequencing reads were filtered for quality using LotuS^109^, and any sequences shorter than 200 base pairs, having average quality scores <20, containing ambiguous base calls, or including mismatches to barcode or sequencing primer were removed. After the sequences were quality filtered and de-multiplexed (barcode and primer sequences removed), reads were pair-end stitched. Resulting sequences were clustered (with a 97% similarity level threshold) into operational taxonomic units (OTUs) using LotuS with the Ribosomal Database Project Classifier^110^. The Ribosomal Database Project Classifier trained on the SILVA v123 reference database^111^ was used to assign taxonomy. Organisms potentially classified to the species level were based on individual OTUs of significance. OTUs failing to classify as bacteria at the kingdom level were removeδαd prior to further analysis of the dataset. Bacterial community profiles were then characterized by the degree of diversity, richness, and relative abundances of the OTUs identified in each sample using QIIME^112^. Output sequences were classified at the phylum and genus levels for comparisons within and across groups. Alpha diversity of microbiota in each treatment group was measured by the Shannon Diversity Index and Operational Taxonomic Unit (OTU) richness was measured by number of observed OTUs. Longitudinal relative abundances of OTUs were used to make phylum and genus-level comparisons within the CONV group of mice (between different postnatal ages and between males and females).

### Fluorescence in situ hybridization (FISH)

For intestinal tissue preparation, the small and large intestines were carefully removed immediately following euthanasia and rapidly dissected. Mouse terminal ileum (3-6 cm above the cecum), cecum, and mouse distal colon were carefully removed, fixed in Carnoy’s fixative (American MasterTech Scientific, Inc) at room temperature for 24 h, then rinsed in 100% ethanol and embedded in paraffin wax. These sectioned tissues were used for FISH staining as described. Four µm sections were mounted on glass slides, baked at 60°C for 1 h, then de-paraffinized with xylene and dehydrated in series from 50% to 100% ethanol. A previously validated, 5′ Texas Red-labelled, *Bifidobacterium* genus-specific probe (Bif164; 5′-CATCCGGCATTACCACCC-3’) targeting the 164-181 bp region of the 16S rRNA gene was used to specifically label bacterial species of the *Bifidobacterium* genus^47^. The probe was hybridized to the samples by adding 15 µL of 2 µM probe to each slide and placing in a 45 °C hybridization chamber for 45 minutes. Nuclei were labeled with DAPI. Intestinal sections from male mice in each cage (total 5-6 per group) were utilized to confirm colonization. The slides were analyzed using a Nikon E600 epifluorescence microscope (Nikon, USA) equipped with appropriate filter set for Texas Red fluorescence (ex 595 nm/em 613 nm).

### Synaptic Plasticity qPCR array

For preparation of RNA for gene expression analysis, the mice were individually anesthetized using isoflurane until no reflex was observed. The mice were rapidly decapitated, and the brains were dissected to isolate the cerebellum, hippocampus, and cortex. The tissues were flash frozen and stored separately at −80 °C until RNA isolation. RNA was isolated from the cerebellum, cortex, and hippocampus of male mice from each developmental timepoint via Trizol. The 260/280 ratio was measured for each sample using a Nanodrop spectrophotometer, and after this step ranged between 1.8 and 2.0. RNA samples were stored at −80°C until use. Two µg of RNA from 5 samples (male mice only) were pooled into one representative sample per treatment, time point, and brain region (27 pooled samples total, n = 5 mice per pooled sample). Pooled RNA was purified with the Zymo RNA clean and concentrate kit according to the manufacturer’s protocol with one modification. Zymo-Spin IIICG columns were used instead of Zymo-Spin IC Columns in order to purify larger quantities of RNA (~100 µg of RNA). A Bioanalyzer (Agilent) was used to verify that the purity and concentration of pooled and cleaned RNA was of sufficient quality and quantity prior to use with the synaptic plasticity qPCR array.

Using the “Mouse Synaptic Plasticity RT² Profiler PCR Array” (PAMM-126Z; Qiagen), 96 genes (Supplementary Table S1; GEO accession #GSE142079) were profiled on the 27 samples (1 pooled sample as described above for each of the 3 brain regions, at each of the 3 time points, in each of the 3 treatment groups). The manufacturer’s protocol was followed for sample preparation. Briefly, 500 ng of each pooled sample was treated with DNase to eliminate genomic DNA contamination, followed by conversion to cDNA using the RT^2^ First Strand Kit. The cDNA was added to the kit mastermix on ice, and distributed to the 384-well plate containing 4 replicate primer assays for the 84 pathway-specific genes. Reactions using SYBR Green chemistry (Qiagen SYBR Green qPCR Mastermix, Cat. no. 330529) were performed in a 384 well block ViiA^TM^ 7 Real-Time PCR system (Applied Biosystems, Foster City, USA). The threshold values were set consistently across all runs in the same analysis (threshold manually set to 0.5 and lower limit of detection set to 35). The resulting table of CT values was uploaded to the data analysis web portal at http://www.qiagen.com/geneglobe. Samples were assigned to control (germ-free) and test groups (conventionalized vs. *Bifidobacterium*-treated), allowing for comparative analysis of gene expression at each time point.

The array contained 84 pathway-focused genes, 5 housekeeping (reference) genes, and a panel of quality control targets to monitor genomic DNA contamination and efficiency of the first strand synthesis and PCR reactions. As defined by the manufacturer’s protocol, the CT value of the PCR efficiency control was within the range of 20 ± 2 for all arrays or samples across runs and between plates. The average values across all 27 samples and 7 plates used in our analysis was CT_PPC_ = 20.37 (std dev ± 0.26). The samples also passed the tests controlling for genomic DNA contamination and first strand synthesis. Gene of Interest (GOI) CT values were normalized using the most stably expressed of the 5 reference genes, which was *Gapdh* on all panels (average *gapdh* CT across all panels = 19.944 (std dev ± 0.33)). The CTs were then sorted into brain region and time point. The fold regulation was calculated using the 2^−ΔCt^ relative quantification, with the threshold for biological significance set to 1.5. The data analysis web portal calculates Fold-change/regulation using ΔΔCT method, in which ΔCT is calculated between gene of interest (GOI) and reference gene (Gapdh), followed by delta-delta CT calculations (delta CT (Test Group)-delta CT (Control Group)). Fold Change was calculated using 2^ (-delta delta CT) formula. Fold-change values greater than one indicates a positive- or an up-regulation, and the fold-regulation is equal to the fold-change. Fold-change values less than one indicate a negative or down-regulation, and the fold-regulation is the negative inverse of the fold-change. Statistical significance between samples was not determined in this analysis due to the pooling of the samples. In addition to the PCR array, we examined gene expression of microglial phagocytic markers by qPCR with primers designed by Primer Design Software (ThermoFisher). Fold-change was calculated using 2^^ΔΔCT^ method, with the reference gene (Gapdh) (n = 8 mice/group). A One-way ANOVA was used to assess significance.

### Assessment of microglial expression of CD11b and CD45 by flow cytometry

Vaporized isoflurane was administered at 1-4% with pressurized oxygen to maintain an appropriate plane of anesthesia during the perfusion. A midline sternotomy was made, and the left ventricle of the heart identified. A 27 x 1/2-gauge needle attached to a pump delivered 5 mL of PBS through the myocardium and into the left ventricle. Simultaneously, a small incision was made to the right atrial appendage to accommodate the additional volume being administered. The mouse was then decapitated, and the brain dissected rapidly on ice. One entire brain hemisphere from each mouse (males, n = 3/group) was placed in ice-cold Dulbecco Modified Eagle’s Medium (DMEM) media (ATCC, cat. 30-2002) containing 10% FBS, and tissue was processed immediately. Red blood cell lysis buffer was then added to deplete any remaining red blood cells for 15 min at 4ºC (RBCs; BD Biosciences). Collagenase/Dispase digestion buffer was added to the isolated cells. The suspension was processed via Percoll gradient to obtain single-cell suspensions of immune cells. These cells were filtered through 100-µm filters, where flow-through is followed with 40-µm filter strains. The cells (10^5^) were blocked with FcR blocker before antibody staining. These single-cell suspensions were stained with live dead staining (APC-Cy7) followed by antibodies (1 µl FITC-conjugated anti-CD45 (eBioscience, cat. 11-0461), 1µl PE-conjugated anti-CD11b (eBiosceince, cat. 12-0110) for 30 min on ice in the dark. Samples (cells) were further washed and fixed in 1% formalin. Multicolor flow cytometry was performed using a BD FACSCanto cell analyzer. Resulting data were collected with the FACSDiva software (BD Biosciences). The accumulated data were analyzed with FlowJo V10 software. IMC’s were quantified with compensation control beads gated for PE, APC and FITC labeled aforementioned antibodies. Forward Scatter (FSC) and Side Scatter (SSC) were performed with unstained isolated primary microglia. Using Flowjo software, the compensation controls (or standards) were applied to all unknown samples. First the cells were selected using forward scatter (FSC) and side scatter (SSC). The selected cell population was gated with FSC on X-axis and Comp-APC-Cy7 (live-dead) on Y axis. The negative-staining live population was further gated with FSC on X-axis and Comp-PE (CD11b^+^) on Y axis. Positive cells were further gated for Comp-PE (CD11b^+^) on X-axis with Comp-FITC (CD45^+^) on y-axis. Cells expressing CD45 were divided into high (hi) or low (lo) expression groups using rectangular gates based on size.

### *In vivo* Purkinje cell electrophysiology

Recordings of Purkinje neurons were performed as previously described^97^. Briefly, *in vivo* extracellular single-unit recordings were obtained using 5-15 MΩ glass electrodes backfilled with 0.9% (w/v) saline. Male mice in GF and BIF groups were used for recordings (*n* = 3-5 animals/group with 3 separate recordings per animal). Animals were anesthetized throughout recording sessions with 0.25-4% isoflurane vaporized in pressurized oxygen and a mixture of 80 mg/kg ketamine and 16 mg/kg dexmedetomidine. Craniotomies were performed 6.5 mm posterior and 0-1.5 mm lateral of Bregma with a diameter of 1-3 mm, permitting access to lobules IV-VIII of the cerebellar vermis. A motorized micromanipulator (Sutter Instruments) was used to lower the electrode into the cerebellum. The signals were band-pass filtered at 0.3-13 kHz, amplified with an ELC-03XS amplifier (NPI), and digitized in real time in Spike2 (CED). Purkinje cell recordings were identified by the presence of both simple spikes and complex spikes, and isolated Purkinje cells were held for ~300 s. Spike sorting and analysis was performed with Spike2 and MATLAB scripts. Spike firing frequency (Hz = spike/s) and firing pattern variability were calculated for each Purkinje cell. Average variability in firing pattern was quantified using the coefficient of variance (CV) of the interspike time interval (ISI) [CV = (standard deviation of ISIs)/(mean of ISIs)]. To measure rhythmicity during burst firing, CV2 of the ISI was calculated [CV2 = 2|ISIn+1 – ISIn| / (ISIn+1 + ISIn)] (Holt *et al*., 1996).

#### Statistical data analysis

Data were analyzed using the GraphPad Prism software (La Jolla, CA, USA). The Kolmogorov-Smirnov test was used to determine if data were normally distributed. Non-parametric data were log-transformed to pass normality tests before analysis by ANOVA. A one-way ANOVA test with appropriate post hoc test was used to evaluate statistical significance. For the flow cytometry analysis, the percentages of cells expressing CD11b^+^ CD45^low^ was compared between groups via one-way ANOVA followed by multiple comparisons testing via Tukey’s Honest Significant Difference test. For the electrophysiology studies, the statistical analyses were performed with unpaired, two-tailed Student’s t-tests. Kruskal-Wallis tests with Dunn’s Multiple Comparison post hoc tests were used to analyze non-parametric data where appropriate. Statistical significance was considered to be *p* < 0.05. All data are expressed as mean ± stdev.

## Supplementary information

Supplemental Table.

## Data Availability

All data generated or analyzed during this study are included in this published article (and its Supplementary Information files). The detailed datasets analyzed during the current study are available from the corresponding author on reasonable request.
