# Supplementary material for: Bifidobacteria shape host neural circuits during postnatal development by promoting synapse formation and microglial function
Source: Sci Rep. 2020 May 8;10:7737. doi: 10.1038/s41598-020-64173-3 (PMC7210968; doi:10.1038/s41598-020-64173-3)
Supplement: Supplementary file 1 — Supplemental Table. [file 41598_2020_64173_MOESM1_ESM.pdf]

SUPPLEMENTARY INFORMATION

Bifidobacteria shape host neural circuits during postnatal development by promoting synapse formation and microglial function

Berkley Luck[1,2,7], Melinda A. Engevik*[1,2], Bhanu Priya Ganesh[3], Elizabeth P. Lackey[2,4], Tao Lin[1,2], Miriam Balderas[2,6], Angela Major[2], Jessica Runge[2,5], Ruth Ann Luna[2,5], Roy V. Sillitoe[1,2,4,5], James Versalovic[1,2,6]

Supplementary Table S1. Synapse-related genes assessed in RT^2 PCR array

RefSeq Number	Symbol	Description
**NM_007399**	Adam10	A disintegrin and metallopeptidase domain 10
**NM_009622**	Adcy1	Adenylate cyclase 1
**NM_009623**	Adcy8	Adenylate cyclase 8
**NM_009652**	Akt1	Thymoma viral proto-oncogene 1
**NM_018790**	Arc	Activity regulated cytoskeletal-associated protein
**NM_007540**	Bdnf	Brain derived neurotrophic factor
**NM_177407**	Camk2a	Calcium/calmodulin-dependent protein kinase II alpha
**NM_178597**	Camk2g	Calcium/calmodulin-dependent protein kinase II gamma
**NM_007664**	Cdh2	Cadherin 2
**NM_009883**	Cebpb	CCAAT/enhancer binding protein (C/EBP), beta
**NM_007679**	Cebpd	CCAAT/enhancer binding protein (C/EBP), delta
**NM_007726**	Cnr1	Cannabinoid receptor 1 (brain)
**NM_133828**	Creb1	CAMP responsive element binding protein 1
**NM_013498**	Crem	CAMP responsive element modulator
**NM_007864**	Dlg4	Discs, large homolog 4 (Drosophila)
**NM_007913**	Egr1	Early growth response 1
**NM_010118**	Egr2	Early growth response 2
**NM_018781**	Egr3	Early growth response 3
**NM_020596**	Egr4	Early growth response 4
**NM_010142**	Ephb2	Eph receptor B2
**NM_010234**	Fos	FBJ osteosarcoma oncogene
**NM_176942**	Gabra5	Gamma-aminobutyric acid (GABA) A receptor, subunit alpha 5
**NM_010305**	Gnai1	Guanine nucleotide binding protein (G protein), alpha inhibiting 1
**NM_008165**	Gria1	Glutamate receptor, ionotropic, AMPA1 (alpha 1)
**NM_013540**	Gria2	Glutamate receptor, ionotropic, AMPA2 (alpha 2)
**NM_016886**	Gria3	Glutamate receptor, ionotropic, AMPA3 (alpha 3)
**NM_019691**	Gria4	Glutamate receptor, ionotropic, AMPA4 (alpha 4)
**NM_008169**	Grin1	Glutamate receptor, ionotropic, NMDA1 (zeta 1)
**NM_010350**	Grin2c	Glutamate receptor, ionotropic, NMDA2C (epsilon 3)
**NM_008172**	Grin2d	Glutamate receptor, ionotropic, NMDA2D (epsilon 4)
**NM_008170**	Grin2a	Glutamate receptor, ionotropic, NMDA2A (epsilon 1)
**NM_008171**	Grin2b	Glutamate receptor, ionotropic, NMDA2B (epsilon 2)
**NM_133442**	Grip1	Glutamate receptor interacting protein 1
**NM_016976**	Grm1	Glutamate receptor, metabotropic 1
**NM_001160353**	Grm2	Glutamate receptor, metabotropic 2
**NM_181850**	Grm3	Glutamate receptor, metabotropic 3

Supplementary Table S1. Synapse-related genes assessed in RT² PCR array (continued)		
**NM_001013385**	Grm4	Glutamate receptor, metabotropic 4
**NM_001081414**	Grm5	Glutamate receptor, metabotropic 5
**NM_177328**	Grm7	Glutamate receptor, metabotropic 7
**NM_008174**	Grm8	Glutamate receptor, metabotropic 8
**NM_152134**	Homer1	Homer homolog 1 (Drosophila)
**NM_010512**	Igf1	Insulin-like growth factor 1
**NM_008380**	Inhba	Inhibin beta-A
**NM_010591**	Jun	Jun oncogene
**NM_008416**	Junb	Jun-B oncogene
**NM_010623**	Kif17	Kinesin family member 17
**NM_013692**	Klf10	Kruppel-like factor 10
**NM_011949**	Mapk1	Mitogen-activated protein kinase 1
**NM_013599**	Mmp9	Matrix metallopeptidase 9
**NM_010875**	Ncam1	Neural cell adhesion molecule 1
**NM_008689**	Nfkb1	Nuclear factor of kappa light polypeptide gene enhancer in B-cells 1, p105
**NM_010908**	Nfkbib	Nuclear factor of kappa light polypeptide gene enhancer in B-cells inhibitor, beta
**NM_013609**	Ngf	Nerve growth factor
**NM_033217**	Ngfr	Nerve growth factor receptor (TNFR superfamily, member 16)
**NM_008712**	Nos1	Nitric oxide synthase 1, neuronal
**NM_008837**	Pick1	Protein interacting with C kinase 1
**NM_016789**	Nptx2	Neuronal pentraxin 2
**NM_008742**	Ntf3	Neurotrophin 3
**NM_198190**	Ntf5	Neurotrophin 5
**NM_008745**	Ntrk2	Neurotrophic tyrosine kinase, receptor, type 2
**NM_021543**	Pcdh8	Protocadherin 8
**NM_008842**	Pim1	Proviral integration site 1
**NM_008872**	Plat	Plasminogen activator, tissue
**NM_021280**	Plcg1	Phospholipase C, gamma 1
**NM_031868**	Ppp1ca	Protein phosphatase 1, catalytic subunit, alpha isoform
**NM_013636**	Ppp1cc	Protein phosphatase 1, catalytic subunit, gamma isoform
**NM_026731**	Ppp1r14a	Protein phosphatase 1, regulatory (inhibitor) subunit 14A
**NM_019411**	Ppp2ca	Protein phosphatase 2 (formerly 2A), catalytic subunit, alpha isoform
**NM_008913**	Ppp3ca	Protein phosphatase 3, catalytic subunit, alpha isoform
**NM_011101**	Prkca	Protein kinase C, alpha
**NM_011102**	Prkcg	Protein kinase C, gamma
**NM_011160**	Prkg1	Protein kinase, cGMP-dependent, type I
**NM_009001**	Rab3a	RAB3A, member RAS oncogene family
**NM_009045**	Rela	V-rel reticuloendotheliosis viral oncogene homolog A (avian)
**NM_011261**	Reln	Reelin
**NM_009061**	Rgs2	Regulator of G-protein signaling 2
**NM_053075**	Rheb	Ras homolog enriched in brain
**NM_019812**	Sirt1	Sirtuin 1 (silent mating type information regulation 2, homolog) 1
**NM_020493**	Srf	Serum response factor
**NM_177340**	Synpo	Synaptopodin
**NM_011593**	Timp1	Tissue inhibitor of metalloproteinase 1

Supplementary Table S1. Synapse-related genes assessed in RT^2 PCR array (continued)		
**NM_013693**	Tnf	Tumor necrosis factor
**NM_007393**	Actb	Actin, beta
**NM_011739**	Ywhaq	Tyrosine 3-monooxygenase/tryptophan 5-monooxygenase activation protein, theta polypeptide
**NM_009735**	B2m	Beta-2 microglobulin
**NM_008084**	Gapdh	Glyceraldehyde-3-phosphate dehydrogenase
**NM_010368**	Gusb	Glucuronidase, beta
**NM_008302**	Hsp90ab1	Heat shock protein 90 alpha (cytosolic), class B member 1
**SA_00106**	MGDC	Mouse Genomic DNA Contamination
**SA_00104**	RTC	Reverse Transcription Control
**SA_00103**	PPC	Positive PCR Control

Supplementary Table S2. Results of Synaptic Plasticity qPCR array (genes >1.5-fold differentially regulated)

CEREBELLUM — P4

CONV vs. GF UP Gene	Fold reg	CONV vs. GF DOWN Gene	Fold reg	BIF vs. GF UP Gene	Fold reg	BIF vs. GF DOWN Gene	Fold reg
Tnf	3.2	*Gabra5*	-6.71	*Tnf*	4.11	*Egr4*	-9.87
Ntf3	2.4	*Bdnf*	-3.85	*Ntf3*	1.93	*Gabra5*	-5.38
Nfkbib	1.89	*Ngf*	-3.49	*Creb1*	1.76	*Bdnf*	-3.72
		Adcy8	-3.37	*Adam10*	1.59	*Ngf*	-3.49
		Egr4	-2.82			*Adcy8*	-3.01
		Mmp9	-2.69			*Grin2d*	-2.59
		Grm8	-2.38			*Nptx2*	-2.55
		Grin2a	-2.23			*Grin2a*	-2.25
		Grin2d	-2.02			*Mmp9*	-2.1
		Camk2g	-1.96			*Camk2g*	-2.07
		Arc	-1.94			*Rab3a*	-1.95
		Nptx2	-1.88			*Ppp1r14a*	-1.94
		Grin1	-1.83			*Arc*	-1.92
		Rab3a	-1.82			*Egr2*	-1.91
		Grm5	-1.76			*Nr4a1*	-1.86
		Grm7	-1.73			*Camk2a*	-1.86
		Gnai1	-1.71			*Grin1*	-1.84
		Camk2a	-1.71			*Grm8*	-1.82
		Adcy1	-1.65			*Egr1*	-1.75
		Ppp1r14a	-1.59			*Adcy1*	-1.74
		Pcdh8	-1.57			*Cebpb*	-1.67
		Nr4a1	-1.56			*Grm5*	-1.66
		Ncam1	-1.5			*Grm7*	-1.63
						Synpo	-1.62
						Fos	-1.6
						Gnai1	-1.56
						Grip1	-1.5

CORTEX — P4

CONV vs. GF UP Gene	Fold reg	CONV vs. GF DOWN Gene	Fold reg	BIF vs. GF UP Gene	Fold reg	BIF vs. GF DOWN Gene	Fold reg
		Bdnf	-2.32	*Arc*	1.87	*Nfkbib*	-2.33
		Kif17	-2.29	*Fos*	1.62	*Tnf*	-2.07
		Gria2	-1.84			*Junb*	-1.77
		Junb	-1.65			*Cdh2*	-1.7
		Mmp9	-1.63			*Homer1*	-1.61
		Creb1	-1.62			*Gria2*	-1.6
		Ntf5	-1.59			*Grm7*	-1.59
		Gabra5	-1.53			*Ntrk2*	-1.56
		Grm8	-1.53			*Creb1*	-1.53
		Prkg1	-1.52			*Klf10*	-1.53
						Ntf3	-1.53
						Grm8	-1.5

HIPPOCAMPUS — P4

CONV vs. GF UP Gene	Fold reg	CONV vs. GF DOWN Gene	Fold reg	BIF vs. GF UP Gene	Fold reg	BIF vs. GF DOWN Gene	Fold reg
Ntf3	1.64	*Plcg1*	-2.73	*Tnf*	1.65	*Ntf5*	-4.24
		Ntf5	-2.1	*Junb*	1.64	*Plcg1*	-3.12
		Prkcg	-1.61			*Grm8*	-1.63
						Ngfr	-1.6
						Pcdh8	-1.56
						Klf10	-1.51

CEREBELLUM — P10

CONV vs. GF UP Gene	Fold reg	CONV vs. GF DOWN Gene	Fold reg	BIF vs. GF UP Gene	Fold reg	BIF vs. GF DOWN Gene	Fold reg
Tnf	8.26	*Grm4*	-1.74	*Tnf*	3.77	*Grin2c*	-1.68
Kif17	2.25	*Grin2c*	-1.64	*Grip1*	1.77	*Bdnf*	-1.62
Gabra5	1.94			*Klf10*	1.75	*Kif17*	-1.53
Adcy8	1.93			*Ngfr*	1.6		
Fos	1.86			*Cdh2*	1.58		
Ntf5	1.84			*Rela*	1.54		
Ngfr	1.67						

CORTEX — P10

CONV vs. GF UP Gene	Fold reg	CONV vs. GF DOWN Gene	Fold reg	BIF vs. GF UP Gene	Fold reg	BIF vs. GF DOWN Gene	Fold reg
Tnf	*4.33*	*Ntf5*	-3.69	*Nos1*	1.88	*Ntf5*	-2.78
Ntf3	*2.19*	*Arc*	-2.02			*Arc*	-1.69
Nos1	*2.03*	*Bdnf*	-1.99				
Grin2d	1.6	*Egr1*	-1.72				
		Ppp1r14a	-1.62				
		Pcdh8	-1.52				

HIPPOCAMPUS — P10

CONV vs. GF UP Gene	Fold reg	CONV vs. GF DOWN Gene	Fold reg	BIF vs. GF UP Gene	Fold reg	BIF vs. GF DOWN Gene	Fold reg
Tnf	3.97	*Arc*	-2.11	*Tnf*	5.07		
Ngfr	1.54	*Ppp1r14a*	-1.76	*Fos*	1.62		
Nos1	1.5	*Egr3*	-1.62	*Adcy8*	1.56		
		Junb	-1.6	*Ntf3*	1.54		
		Grin2c	-1.56				

Supplementary Table S2. Results of Synaptic Plasticity qPCR array (genes >1.5-fold differentially regulated) (Continued)

CEREBELLUM							
P20							
CONV vs. GF				BIF vs. GF			
UP		DOWN		UP		DOWN	
Gene	Fold reg	Gene	Fold reg	Gene	Fold reg	Gene	Fold reg
Nfkbib	3.06	*Plat*	-3.84	*Tnf*	2.33	*Gabra5*	-2.01
Arc	1.54	*Fos*	-3.41	*Adcy8*	2.01	*Ntf5*	-3.34
Grin2d	1.52	Cebpd	-2.61	*Nfkbib*	1.69		
		Nr4a1	-2.32	*Pcdh8*	1.69		
		Egr1	-1.55	*Fos*	1.67		
				Grin2b	1.65		
				Egr3	1.64		
				Egr4	1.57		
				Grm2	1.52		

CORTEX							
P20							
CONV vs. GF				BIF vs. GF			
UP		DOWN		UP		DOWN	
Gene	Fold reg	Gene	Fold reg	Gene	Fold reg	Gene	Fold reg
Adcy8	*7.07*	*Arc*	*-2.42*	*Adcy8*	6.34	*Ntf5*	-1.62
Gria2	*1.8*	*Ntf5*	*-1.99*	*Tnf*	2.33		
Grm3	*1.69*	*Cebpd*	-1.72	*Bdnf*	2.05		
Junb	*1.66*	*Egr2*	-1.59	*Egr3*	1.85		
Adam10	*1.55*			*Ppp1cc*	1.82		
Inhba	*1.52*			*Grm3*	1.81		
				Inhba	1.8		
				Homer1	1.78		
				B2m	1.76		
				Gria2	1.72		
				Pcdh8	1.71		
				Adam10	1.66		
				Sirt1	1.64		
				Ppp3ca	1.59		

HIPPOCAMPUS							
P20							
CONV vs. GF				BIF vs. GF			
UP		DOWN		UP		DOWN	
Gene	Fold reg	Gene	Fold reg	Gene	Fold reg	Gene	Fold reg
Ntf5	2.17	*Pcdh8*	-1.68	*Fos*	1.85	*Ppp1r14a*	-1.73
		Plat	-1.67			*Timp1*	-1.51
		Timp1	-1.53				
		Creb1	-1.52				
		Cnr1	-1.5				

The fold regulation was calculated using the $2^{-\Delta Ct}$ relative quantification method, with the threshold for biological significance set to 1.5.

Supplementary Table S3. Results of qRT-PCR indicating downregulation of synaptic genes in adult *Bifidobacterium* mono-colonized mice

Gene	Expression in *B. dentium* mono-associated mice relative to germ-free mice	
	Males	Females
Synaptophysin (*Syp*)	↓*	↓
Post-synaptic density protein 95 (*Psd-95/dlg4*)	↓↓	↓
Neurogranin (*Nrgn*)	↓	**NC**
Synapsin 1 *(Syn1)*	↓↓	↓
Reelin (*Reln*)	↓↓**	↓**
Neuroligin2 *(Nlgn2)*	↓↓***	**NC**
GABA Receptor B1 (*Gabbr1*)	↓↓*	↓

↓/ ↑ = less than 2-fold expression difference

↓↓ /↑↑= greater than 2-fold expression difference

*p<0.05, **p<0.01, ***p<0.001

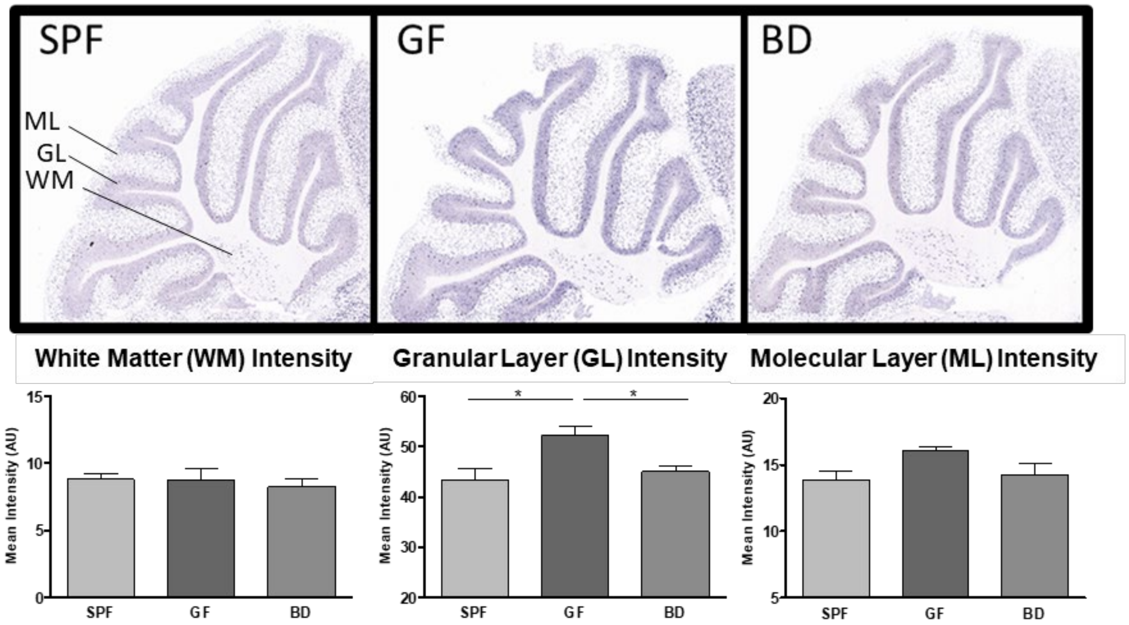

Supplemental Figure S1. RNA *in situ* hybridization demonstrates downregulated expression of the synaptic gene *dlg4* in the adult mouse brain.

(Top panels) Representative plots of RNA in situ hybridization demonstrating *dlg4* expression within the cerebellum of adult male germ-free and *B. dentium*- mono-associated mice. (Bottom panel) Plots are a semi-quantitative analysis of *dlg-4* expression in each cerebellar layer obtained by measuring mean colorimetric intensity in each region of interest via ImageJ. All data are presented as means ± SEM. *p<0.05, One-way ANOVA, Tukey HSD. (n=5 adult mice per group) SPF = Specific Pathogen Free, GF=Germ-free, BD=*B. dentium* mono-associated.
